# Effect of Dry Oxidation on the Optical Response and Morphology of Mesoporous Hybrid Structures

**DOI:** 10.1021/acsomega.5c01186

**Published:** 2025-10-22

**Authors:** María R. Jiménez-Vivanco, Miller Toledo-Solano, Raúl Herrera, Maricela Santana, Eduardo Lugo

**Affiliations:** † Instituto de Física, Universidad Nacional Autónoma de México (UNAM), Circuito de la Investigación Científica, Ciudad Universitaria, Mexico City, 04510 Mexico City, Mexico; ‡ CONAHCYT-Facultad de Ciencias Físico-Matemáticas, Benemérita Universidad Autónoma de Puebla, Av. San Claudio y Av. 18 Sur, Col. San Manuel, Ciudad Universitaria, 72570 Puebla, Mexico; § Laboratorio de Biología Periodontal, División de Estudios de Posgrado e Investigación Facultad de Odontología, Universidad Nacional Autónoma de México, Mexico City 04510, Mexico; ∥ Faubert Lab, School of Optometry, 5622 University of Montreal, Montreal QC H3T 1P1, Canada; ⊥ Sage-Sentinel Smart Solutions, 1919-1 Tancha, Onna-son, Kunigami-gun, Okinawa 904-0495, Japan; # Facultad de Ciencias Físico-Matemáticas, Ciudad Universitaria, Puebla 72570, Puebla, Mexico

## Abstract

This work presents a detailed experimental and theoretical investigation of periodic and quasiperiodic hybrid photonic structures composed of porous silicon (PS) and thermally oxidized porous Si–SiO_2_. Designed with a Fibonacci sequence and embedded between asymmetric Bragg mirrors, the structures were fabricated via electrochemical etching on p-type (100)-oriented silicon wafers with distinct doping levels (P^+^ and P^++^). A two-step dry oxidation process (350 °C and 800 °C) was employed to stabilize the porous network and transform PS into a robust hybrid Si–SiO_2_ matrix. SEM and EDS analyses revealed that wafer doping significantly affects morphology, oxide growth, and silicon retention, with P^+^-based structures maintaining smoother surfaces and higher Si content postoxidation. Optical transmission spectra revealed that oxidation induces substantial blue shifts in localized defect modes, resulting from changes in refractive index and optical path length. Notably, porous Si–SiO_2_ structures fabricated from P^+^ wafers exhibit sharper and less attenuated localized modes compared to those from P^++^ wafers, due to reduced Rayleigh scattering losses. Scattering loss estimations corroborate these findings. This study uniquely correlates morphology, doping, and oxidation kinetics to optical performance, demonstrating that dry oxidation can be strategically employed to enhance light confinement and reduce optical losses in mesoporous Fibonacci-based photonic structures. These results position porous Si–SiO_2_ hybrid systems as promising platforms for low-loss photonic devices, sensors, and microcavity-based applications.

## Graphical abstract

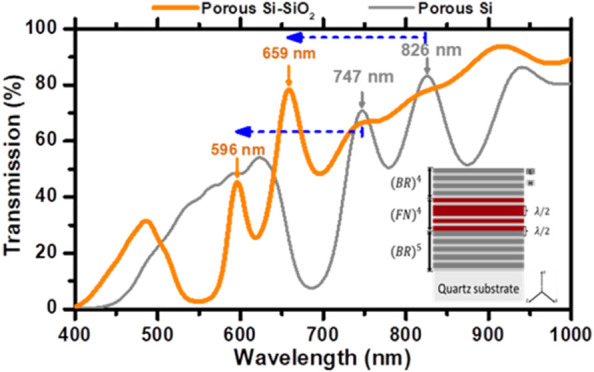

## Introduction

Porous silicon (PS) has been extensively used in the fabrication of photonic structures due to its low-cost production and high tunability in optical and structural properties.^1^ The most widely adopted technique for PS fabrication is the electrochemical anodization of silicon wafers in HF-ethanol-based electrolytes,^2^ which enables precise control over layer thickness, porosity, and pore diameter.^3,4^ As a result, diverse photonic structures, such as Bragg reflectors (BR),^5^ rugate filters,^6^ microcavities,^7^ and aperiodic systems like Fibonacci structures (FN),^8^ can be fabricated using this approach.

Among these, quasiperiodic structures (QS), including Fibonacci, Thue-Morse, Cantor, Rudin-Shapiro, and double-periodic sequences, have attracted significant interest due to their unique photonic properties, which exist between periodic order and complete randomness.^9,10^ These structures display pseudobandgaps and critical modes linked with their long-range order and lack of translational symmetry.^11,12^ Such spectral features enable custom light-matter interactions in compact platforms, supporting applications in random lasers,^13^ dense wavelength division multiplexing (DWDM),^14^ pressure sensing,^15^ near-IR sensors,^16^ gas sensing,,^17^ chemical sensing,^18^ biosensor,^19^ ultrasensitive molecule detection,^20^ γ radiation detector,^21^ logic gates and optical modulators,^22,23^ and antireflection coatings for solar cells.^24^

Although QS have been extensively studied in dielectric multilayers, few experimental reports explore these structures fabricated with PS. Some theoretical works have modeled the influence of the angle of incidence on photonic bandgap formation in periodic and quasiperiodic PS structures, including Fibonacci and Thue-Morse lattices, as well as hybrid BR-FN-BR configurations.^25^ Others have examined time-resolved transmission properties and band-edge excitation effects in Fibonacci PS structures, showing phenomena such as pulse stretching and coherent beatings.^8,26^ Cantor-based PS QS have also been proposed for gas sensing and refractive index modulation, showing improvements in sensitivity and line width.^27–29^ Recent work into porous-Si Fibonacci-conjugated arrays has uncovered ultrastrong coupling and quasi-bound-states-in-the-continuum (quasi-BIC), including room-temperature polariton repulsion in CdSeS/Zn quantum-dot hybrids.^30^ These findings underscore the photonic richness of quasiperiodic architectures and suggest promising new directions in light–matter interaction that our current work could also explore.

Despite these advances, experimental realizations of PS-based QS face challenges due to optical losses, which degrade device performance. Optical losses, particularly absorption and scattering, are significant limitations in photonic structures based on PS. PS absorbs strongly in the UV due to defect states and quantum confinement. At the same time, scattering arises from surface roughness, pore wall irregularities, and internal inhomogeneities.^31,32^ These losses critically reduce the quality factor (Q) in microcavities, negatively affecting lasing thresholds and optical feedback.^33–39^ Furthermore, scattering is strongly dependent on morphology, which is influenced by the type of silicon wafer (P^+^, P^++^), doping level, crystalline orientation, and etching conditions.^40–45^ For example, mesoporous structures etched in N^+^/P^+^ wafers tend to form branched ⟨100⟩-oriented pores, while variations in doping concentration and etching time impact charge transport and porosity.^46,47^

Recent studies have expanded dry-oxidation strategies to porous quartz-based systems, showing superior thermal and environmental stability compared to PS multilayers. In ref 42, it is reported that oxidized porous quartz maintained structural integrity and photonic bandgap positions up to 450 °C, unlike PS-based structures, which exhibit 10 nm redshifts. This result underscores the enhanced robustness of porous Si–SiO_2_ frameworks under extreme conditions.^30,48^ Additionally, hybrid configurations that integrate metal nanoparticles or quantum dots into PS microcavities have garnered attention. For example, Ag-nanoplatelet-enhanced PS cavities demonstrated substantial fluorescent enhancement,,^20,49^ and the deterministic integration of SiO_2_ cladding layers in photonic circuits has recently enabled low-loss thermo-optic tuning in integrated devices.^50^

In our previous work, we fabricated symmetric PS and porous SiO_2_ quasiperiodic microcavities to explore strategies for reducing optical losses. In the first study,^25^ we used P^++^ wafers to create structures with a (BR)_6_–(FN)_4_–(BR)_6_ layout. These devices showed dual localized modes at 550 and 594 nm, which shifted to 393 and 432 nm after oxidation. A preliminary morphological analysis was also included. In a subsequent study,^32^ we used P^+^ wafers and a revised layout, (BR)_5_–(FN)_4_–(BR)_6_, to decrease bottom reflector thickness. The localized modes shifted from 587 and 639 nm to 470 and 518 nm after oxidation. Although porous SiO_2_ microcavities in both cases exhibited reduced scattering losses (∼50%) and minimal absorption (∼0.5%), the morphology parameters were borrowed from unrelated studies, possibly underestimating actual scattering losses.

To overcome these limitations, we present a novel experimental realization of asymmetric PS and porous Si–SiO_2_ hybrid microcavities centered around a Fibonacci quasiperiodic core. This work marks the first comprehensive report combining (i) quasiperiodic Fibonacci structures with asymmetric Bragg reflectors, (ii) detailed morphological and compositional analysis obtained directly from the fabricated samples, (iii) experimental absorption losses analysis, and (iv) quantum-mechanical Rayleigh scattering modeling based on experimental morphology. It introduces several innovations, including an asymmetric microcavity configuration. We designed and fabricated a (BR)_4_–(FN)_4_–(BR)_5_ microcavity structure, where symmetry breaking is achieved by altering the order and thickness of the Bragg mirrors. Although theoretical predictions suggest that asymmetry can improve field localization and transmittance in multilayered photonic structures,^51^ experimental validations in porous QS systems have been lacking. Here, we experimentally verify these effects, further reducing the bottom reflector thickness to minimize losses.

Regarding material variation using P^+^ and P^++^ wafers: To assess how dopant concentration and wafer resistivity influence morphology and scattering, we used two substrate types with resistivities of 0.01–0.02 Ω·cm (P^+^) and 0.001–0.005 Ω·cm (P^++^). Detailed morphological analysis with SEM imaging using secondary and backscattered electrons to measure key parameters, such as pore diameter, interpore spacing, wire radius, and branch length. P^+^ wafers produced flat mesoporous surfaces, whereas P^++^ wafers resulted in spherical mesoporous structures. Simultaneous measurement of absorption and scattering losses was performed: absorption was directly measured. In contrast, scattering losses were modeled using the quantum-mechanical Rayleigh scattering framework proposed by Solano et al.^52,53^ Accurate modeling depended on morphology data specific to each sample, as scattering is highly sensitive to pore geometry and surface irregularities. Our results show that oxidized hybrid microcavities fabricated on P^+^ wafers exhibit significantly lower scattering losses compared to those on P^++^ wafers. Chemical composition profiling via energy-dispersive X-ray spectroscopy (EDS) was used to analyze silicon and oxide content before and after oxidation. Dry oxidation increased the oxide volume, resulting in thinner pores and thicker wire regions. Optical characterization and modeling: Transmission spectra indicated that porous Si–SiO_2_ hybrid microcavities enhance optical throughput and produce narrower photonic bandgaps, especially in devices fabricated on P^+^ wafers. These experimental findings were validated with transfer matrix method simulations that incorporated variations in refractive index due to oxidation and changes in porosity.

By optimizing both the material composition (oxidized porous Si–SiO_2_) and architectural design (asymmetry and quasiperiodicity), this study advances the reduction of optical losses in photonic crystal (PS)- based microcavities. It expands their applicability in ultraviolet photonics, sensing, and integrated photonic systems.

## Results and Discussion

This work first demonstrates that hybrid porous silicon (PS) and porous Si–SiO_2_ structures fabricated using P^+^ and P^++^ silicon wafers exhibit distinct morphological characteristics. To explore these differences, two hybrid PS structures were fabricated: one using a P^+^ wafer and the other with a P^++^ wafer, both oriented in the (100) crystallographic direction. X-ray diffraction (XRD) analysis was conducted to confirm this crystal orientation, as detailed in the Methods section of the Supporting Information.

The first PS hybrid structure, based on the P^+^ wafer, was fabricated using alternating current density pulses of 5 mA and 80 mA with anodization times of 1.00 and 4.98 s, respectively, to form alternating low- and high-porosity layers. A high-resolution SEM image of this structure is shown in Figure 1a. Similarly, the second hybrid structure was prepared on a P^++^ wafer using the same current densities but with anodization times of 1.16 and 7.28 s. The resulting morphology is shown in Figure 1c.

**Figure 1:**
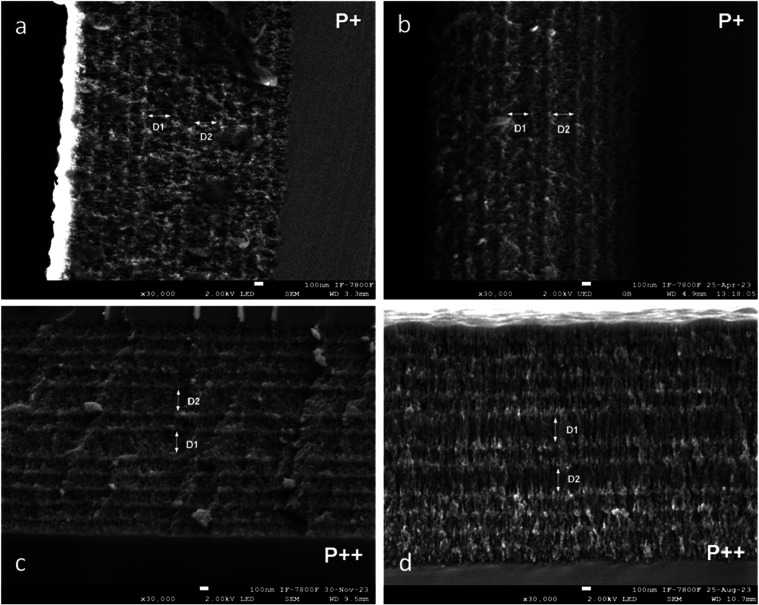
Cross-sectional secondary electron SEM images of hybrid photonic structures. (a) PS-based hybrid structure fabricated using P^+^ wafers. (b) Porous Si–SiO_2_ hybrid structure obtained after oxidation of the sample in (a). (c) PS-based hybrid structure fabricated using P^++^ wafers. (d) Porous Si–SiO_2_ hybrid structure derived from the sample in (c) after oxidation. All structures consist of 26 alternating layers of low (*n*_L_) and high (*n*_H_) refractive index. The contrast in the images highlights the porosity difference, with dark and light gray corresponding to high- and low-porosity layers, respectively.

Both PS hybrid structures were subjected to a two-step dry oxidation process in ambient air to produce porous Si–SiO_2_ hybrid structures. The first stage consisted of a low-temperature treatment at 350 °C for 30 min, which stabilized the PS matrix and prevented structural collapse during the subsequent high-temperature step. The second stage involved oxidation at 800 °C for 30 min to transform the PS into porous Si–SiO_2_. The resulting morphologies are shown in Figure 1b (for P^+^) and Figure 1d (for P^++^).

As shown in Figure 1a–d, both hybrid structures comprise 26 alternating layers of low (*n*_L_) and high (*n*_H_) refractive index. The defect layers (D1 and D2) are identifiable, having twice the thickness of the high-porosity layers. In the SEM images, the high-porosity layers appear dark gray, while the low-porosity layers appear light gray. After dry oxidation, the number of layers is preserved. Still, each layer becomes thicker due to the expansion of the porous matrix as silicon is partially converted into Silicon Dioxide and air voids are partially filled by oxide growth, resulting in more mechanically stable structures.

Table 1 summarizes the layer thicknesses measured from SEM images before and after oxidation. Here, *d*_*L*_ and *d*_*H*_ refer to the thicknesses of low- and high-refractive index layers, respectively. It is essential to note that the thickness of each PS layer correlates linearly with the anodization time, enabling fine control over layer dimensions through adjustments to the pulse durations.^2,54^ As shown in Table 1, the postoxidation thicknesses of all layers exceed their preoxidation values due to the formation and expansion of the SiO_2_ network within the porous structure.^1^

**Table 1:** Individual Thicknesses Obtained by SEM of the Hybrid Structures

PS hybrid structures	thickness before dry oxidation	porous Si-SiO_2_ hybrid structures	thickness after dry oxidation
figure a	*d*_L_ = 131.4 nm	figure b	*d*_L_ = 133.4 nm
	*d*_H_ = 48.35 nm		*d*_H_ = 50.35 nm
figure c	*d*_L_ = 120 nm	figure d	*d*_L_ = 122 nm
	*d*_H_ = 53 nm		*d*_H_ = 55 nm

To further investigate surface morphology, SEM images of the top surfaces of the PS (Figure 2a,c) and porous Si–SiO_2_ (Figure 2b,d) structures were analyzed. Figure 2a,b show the surface morphologies of structures fabricated on P^+^ wafers before and after oxidation, respectively. In both cases, a flat and uniform porous texture is observed. In contrast, structures fabricated on P^++^ wafers (Figure 2c,d) exhibit a rougher and more irregular porous surface, both before and after oxidation. These results confirm that while dry oxidation preserves the porous nature of the structures, the underlying wafer type has a significant influence on their morphology.

**Figure 2:**
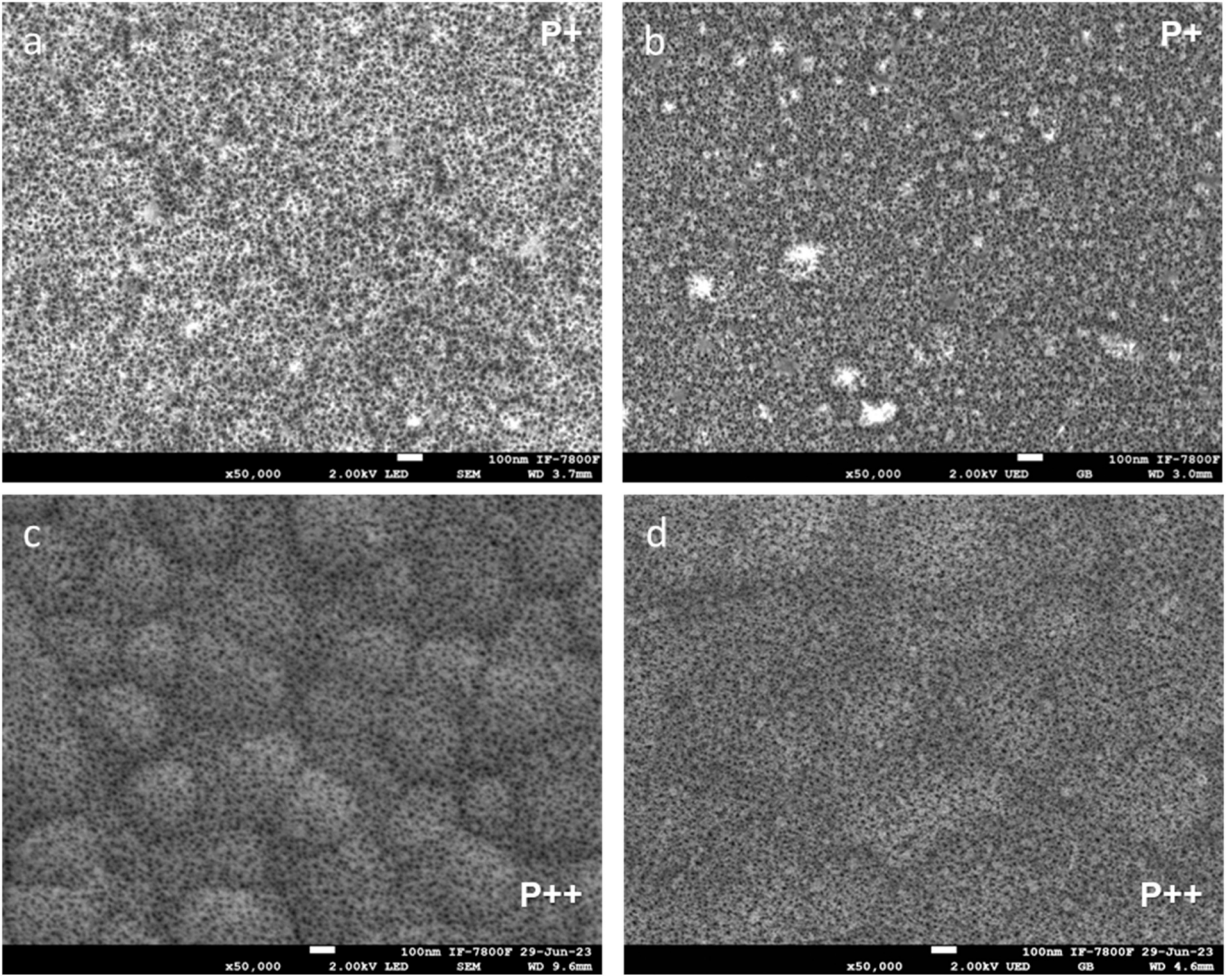
Surface-section secondary electron SEM images of hybrid structures. (a) PS-based hybrid structure fabricated with P^+^ wafers. (b) Porous Si–SiO_2_ structure derived from the oxidation of (a). (c) PS-based hybrid structure fabricated with P^++^ wafers. (d) Porous Si–SiO_2_ structure obtained after oxidation of (c). All images reveal the porous network morphology on the surface. Samples fabricated with P^+^ wafers exhibit smoother, flatter surfaces, while those using P^++^ wafers show rougher textures, indicating a morphology-dependent evolution during oxidation.

Previous studies have established that the anodization current density has a greater impact on PS morphology than anodization time and is therefore considered a critical parameter for controlling surface uniformity.^55^ Specifically, lower current densities tend to produce surfaces with more pronounced protrusions. In contrast, higher current densities promote smoother morphologies.^56^ Additionally, surface roughness in PS has been shown to increase with anodization time initially but tends to decrease with prolonged etching.^46^ The resulting morphology also depends on several other factors, including the crystallographic orientation of the silicon wafer, the anodization regime, the configuration of the electrolytic cell, and any pre- or postprocessing treatments applied to the sample.^41^ Recent work further shows that adding oxidant mixtures (e.g., H_2_O_2_) during anodization can thin pore walls and reduce surface roughness in high-porosity PS films, as revealed by spectroscopic ellipsometry and SEM.^57^

Moreover, the surface of PS has been reported to become smoother after thermal oxidation,^50,53^ which explains why the oxidized structure shown in Figure 2d appears smoother than its unoxidized counterpart in Figure 2c. Despite these morphological changes, both porous Si–SiO_2_ structures (Figure 2b,d) retain their porous character and overall structural integrity following the oxidation process. Thermal oxidation has repeatedly been shown to smooth PS surfaces.^55,58^ Notably, in situ thermal oxidation studies indicate that at 800 °C, PS undergoes silica formation that uniformly passivates pore walls and eliminates microscale asperities, effectively lowering the RMS roughness by ≥30%.^58^ Detection by ellipsometry and FTIR confirms these morphological and compositional transformations.^58^ Despite these changes, both porous Si–SiO_2_ samples maintain their porous nature and layer periodicity after oxidation (Figure 2b,d), yielding mechanically stabilized structures with improved surface quality.

Figure 3 presents energy-dispersive X-ray spectroscopy (EDS) measurements used to quantify the elemental composition of both PS and porous Si–SiO_2_ structures. In Figure 3a,c, the EDS spectra of PS samples fabricated on P^+^ and P^++^ wafers indicate that silicon dominates the composition, with only minor oxygen content. Specifically, for PS formed on P^+^ wafers (Figure 3a), the silicon mass fraction is 97.21%, with oxygen at 2.79%. PS formed on P^++^ wafers (Figure 3c) exhibits a similar trend, with 95.93% silicon and 4.07% oxygen. These low oxygen levels are consistent with native oxidation occurring on porous surfaces upon exposure to air, confirming minimal oxidation during fabrication.

**Figure 3:**
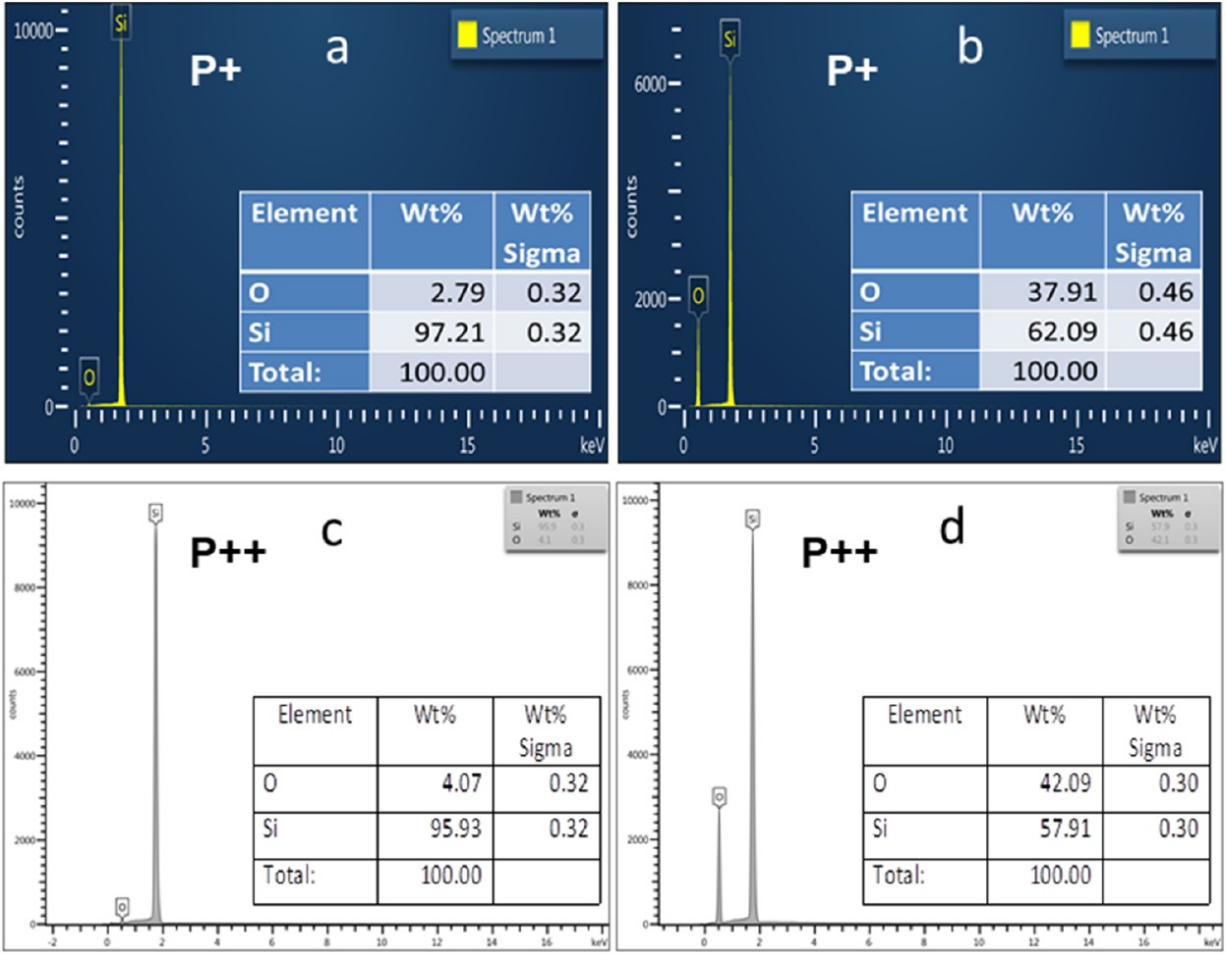
EDS elemental spectra of hybrid structures before and after oxidation. (a) PS hybrid structure fabricated with P^+^ wafers. (b) Porous Si–SiO_2_ hybrid structure obtained by dry oxidation of (a). (c) PS hybrid structure fabricated with P^++^ wafers. (d) Porous Si–SiO_2_ hybrid structure obtained by dry oxidation of (c). Each spectrum includes a compositional table showing the atomic percentages of Si and O. The porous Si–SiO_2_ structures were produced via a two-step dry oxidation in air: 350 °C for 30 min to stabilize the PS matrix, followed by oxidation at 800 °C for 30 min in an electric muffle furnace.

Following the two-step dry oxidation process, the EDS spectra of the resulting porous Si–SiO_2_ structures (Figure 3b,d) show a notable increase in oxygen content. For samples based on P^+^ wafers, the silicon and oxygen mass fractions shift to 62.09 and 37.91%, respectively (Figure 3b). Structures formed on P^++^ wafers display slightly more oxidation, with 57.91% silicon and 42.09% oxygen (Figure 3d). These results indicate significant conversion of Si into SiO_2_, supporting the effectiveness of the oxidation process in altering the porous matrix.

For comparison, stoichiometric SiO_2_ consists of approximately 47% Si and 53% O by mass.^32^ Thus, the oxidized P^+^-based structures retain about 15.09% excess silicon, while the P^++^-based samples retain 10.91%. This residual silicon may be associated with incomplete oxidation or the presence of suboxide species, such as SiO*x* (1 < *x* < 2), which are commonly reported in thermally oxidized PS structures.^1,32,54^

EDS analysis is a widely used method for monitoring compositional changes in oxidized porous structures. Recent studies have demonstrated that the oxidation process can lead not only to changes in the Si:O ratio but also to structural densification and shifts in the refractive index, depending on the porosity and pore morphology.^52,53^ These findings are consistent with our observations and highlight the role of wafer type and morphology in controlling the degree of oxidation.

Figure 4 presents pore size distribution histograms for the hybrid structures fabricated using P^+^ and P^++^ wafers. The data were obtained through analysis of SEM images captured using backscattered electrons from the superficial cross-section, as shown in the insets (Figure 4a’,b’,c’,d’). Pore diameters were measured using *ImageJ* software. For the PS structures, the pore diameters ranged from 5 to 30 nm, with average values of 19 ± 6 nm and 16 ± 6 nm for samples fabricated with P^+^ and P^++^ wafers, respectively. After dry oxidation, the average pore diameters in the resulting porous Si–SiO_2_ structures decreased slightly to 15 ± 8 nm (P^+^ wafers) and 14 ± 3.5 nm (P^++^ wafers).

**Figure 4:**
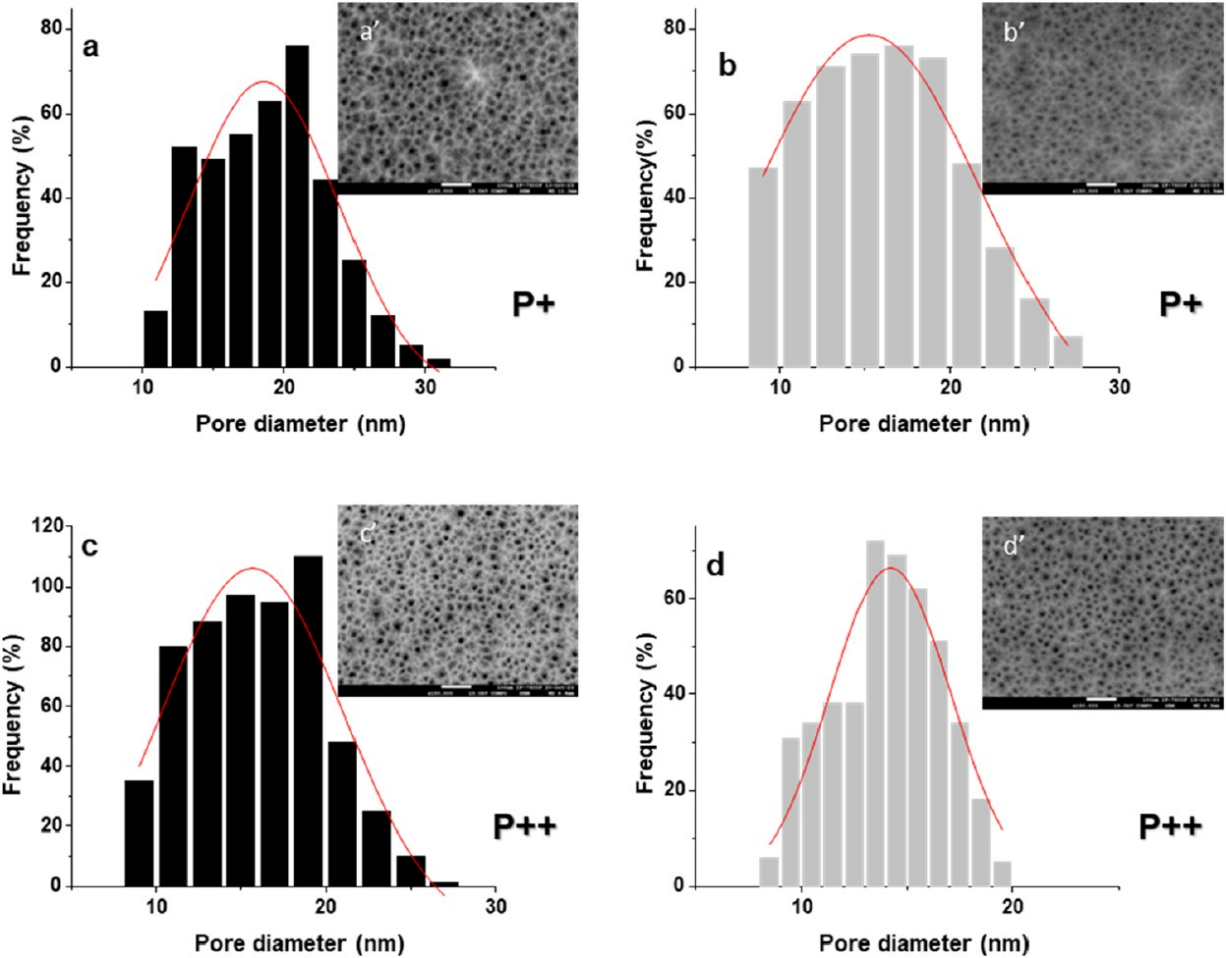
Pore diameter distribution histograms and corresponding backscattered electron SEM images of the superficial sections. (a, c) Pore size distribution of PS hybrid structures fabricated with P^+^ and P^++^ wafers, respectively. (b, d) Pore size distribution of porous Si–SiO_2_ hybrid structures obtained after dry oxidation of the samples shown in (a) and (c), respectively. The insets display the backscattered electron SEM images used to extract pore diameter data. The average pore diameters were 19 ± 6 and 16 ± 6 nm for PS samples fabricated with P^+^ and P^+^ wafers, respectively, and 15 ± 8 and 14 ± 3.5 nm for porous Si–SiO_2_ samples fabricated with P^+^ and P^++^ wafers, respectively.

Figure 4a,c display the pore size distributions for the unoxidized PS structures fabricated with P^+^ and P^++^ wafers, respectively, while Figure 4b,d correspond to the oxidized porous Si–SiO_2_ structures. In all samples, the pores appear relatively uniformly distributed, with an average center-to-center pore spacing of approximately 29 nm across the structures, as observed in the SEM insets. It is also noted that in PS structures, the pore spacing tends to decrease with increasing porosity, consistent with previously reported trends.^47^

Table 2 summarizes the measured pore and wire diameters of both PS and porous Si–SiO_2_ structures. A clear trend is observed: after dry oxidation, the average pore diameter decreases, while the wire diameter increases. This outcome results from the oxidation process, during which a portion of the silicon is converted into SiO_2_, thereby thickening the silicon skeleton and partially filling the initially occupied void spaces with air. The oxidation temperature and duration primarily determine the extent of SiO_2_ growth on PS wires.^1^

**Table 2:** Pore Diameter and Wire Size of PS and Porous Si-SiO_2_ Structures

wafer	pore diameter before dry oxidation (nm)	PS wire diameter (nm)	pore diameter after dry oxidation (nm)	porous Si-SiO_2_ wire diameter (nm)
P+	19 ± 6	12 ± 2.5	15 ± 8	13 ± 2.5
P++	16 ± 6	10 ± 2	14 ± 3.5	12.5 ± 2

In PS structures, pore size is chiefly influenced by the applied current density during anodization, the electrolyte composition, and the resistivity of the silicon wafers (SWs).^4,47,59^ It has been established that mesoporous Si, with pore diameters ranging from 5 to 50 nm, is typically formed using highly doped SWs as a substrate,^4^ which is consistent with the pore sizes obtained in our study. Furthermore, the pore size remains within the mesoporous regime even after oxidation in both P^+^ and P^++^-based porous Si–SiO_2_ structures.

Controlling the pore size in PS is particularly relevant for applications such as biosensing, where specific pore diameters can either enhance analyte sensitivity or serve as molecular filters by restricting the entry of larger species.^60^

The SEM results (Figure 1) and pore size distribution histograms (Figure 4) provide valuable insights into postoxidation morphological changes. The reduction in pore diameters and increase in layer thickness postoxidation are well-documented, but exploring their impact on mechanical stability would be interesting. The effect on the mechanical stability of the PS and oxidized PS layer at low temperature has been reported. As a result, oxidation improved the mechanical properties of oxidized PS layers, with an increase in hardness and Young’s modulus compared to the PS layer. This improved behavior is primarily attributed to a layer of SiO_2_ that forms at the columnar surface of PS, resulting in the densification of the entire structure and the encapsulation of the nanocrystallites that comprise the columnar structure. Therefore, oxidation reduces the formation and growth of cracks. Additionally, the formation of oxide improved the mechanical stability of the structure while maintaining good thermal and dielectric properties.^61^ Moreover, it has been observed that the effects on the mechanical properties of PS oxidized at high (850 °C for 4 h) and low (300 °C for 2 h) oxidation temperatures. Hardness and Young’s modulus of oxidized PS and PS have been compared, where the lowest Young’s modules and hardness (19.5 and 0.9 GPa) were observed in PS, whereas it was higher for oxidized PS at 850 °C for 4 h (39.3 and 3.3 GPa) and 300 °C for 2 h (29.6 and 1.6 GPa).^62^ In the future, it would be interesting to compare the mechanical properties of PS and oxidized PS multilayers; we would expect to obtain a higher Young’s modulus and hardness for oxidized PS multilayers.

Figure 5 shows the theoretical (black line) and experimental (red line) transmission spectra of hybrid structures of PS and porous Si–SiO_2_ obtained with P^+^ (Figure 5a,c) and P^++^ (Figure 5b,d) wafers. Both PS structures were designed to exhibit two localized modes at 747 and 826 nm (Figure 5a,b), where PS is considered a material with low optical losses due to absorption. However, absorption losses were accounted for in the theoretical transmission spectra since our structures do not exhibit perfect (100%) transmittance; they reflect, absorb, and transmit light. Theoretical transmission spectra of the PS structures were fitted using high (66 and 62%) and low porosities (46 and 45%) to determine each PS layer’s refractive index.

**Figure 5:**
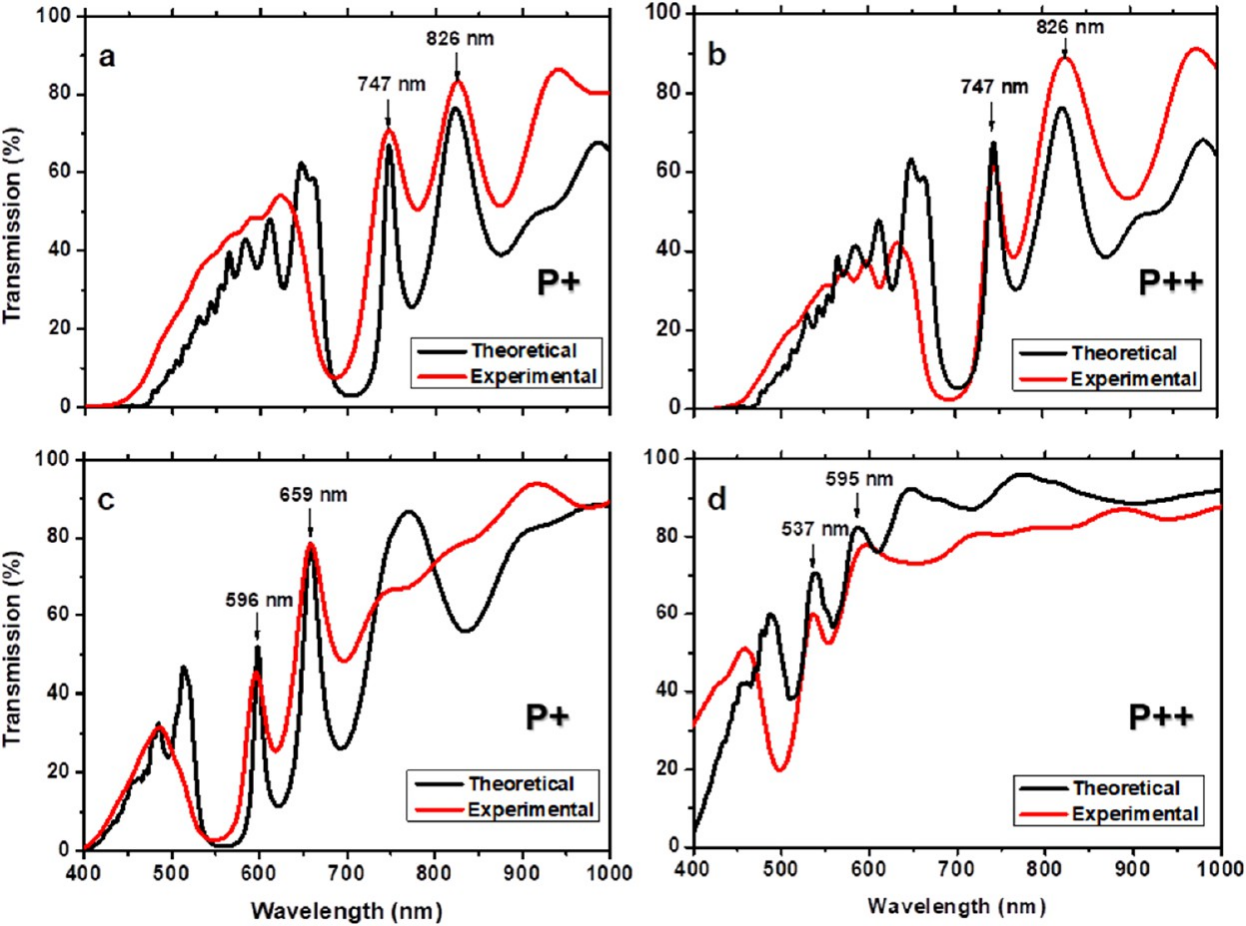
Theoretical (black lines) and experimental (red lines) transmission spectra of PS and porous Si–SiO_2_ hybrid structures fabricated with P^+^ and P^++^ wafers. (a, b) Transmission spectra of PS hybrid structures obtained with P^+^ (a) and P^++^ (b) wafers, designed to exhibit two localized modes. (c, d) Transmission spectra of the same structures after two-stage dry oxidation, forming porous Si–SiO_2_ hybrid structures using P^+^ (c) and P^++^ (d) wafers. Shifts in the localized modes are attributed to changes in the refractive index and a reduction in porosity following oxidation.

Afterward, both structures were subjected to a two-stage dry oxidation process. As a result, the localized modes of the porous Si–SiO_2_ structures fabricated on P^+^ wafers shifted to shorter wavelengths at 596 and 659 nm (Figure 5c), and those manufactured on P^++^ wafers moved to 537 and 595 nm (Figure 5d). Theoretical transmission spectra of both oxidized structures were simulated using the transfer matrix method as in previous work.^10,31,32^ We employed high porosities (40 and 34%) and low porosities (20 and 7%) to calculate the effective refractive index of each porous Si–SiO_2_ layer. The wavelength shift in localized modes is attributed to a reduction in the complex refractive index and a decrease in the optical path length (n·d), resulting from oxidation-induced structural changes (see Tables 1, 3, and 4).

**Table 3:** Hybrid Structures Parameters using P+ Wafers

the refractive index of PS	refractive index of porous Si-SiO_2_	P(%)	P_ox_(%)	F_ox_(%)	F_si_(%)	
n_L_ = 1.94–0.0037*i*	*n*_L_ = 1.476–0.0024*i*	66	40	52	34	8
*n*_H_ = 2.435–0.0053*i*	*n*_H_ = 1.98–0.0063*i*	46	20	54	54	26

**Table 4:** Hybrid Structures Parameters Using P++ Wafers

the refractive index of PS	refractive index of porous Si-SiO_2_	P(%)	Pox(%)	Fox(%)	Fsi(%)	
*n*_L_ = 2.037–0.004*i*	*n*_L_ = 1.544–0.0047*i*	62	34	57	38	9
*n*_H_ = 2.458–0.0054*i*	*n*_H_ = 1.423	45	7	93	55	0

As shown, the porous Si–SiO_2_ structure, fabricated with P^++^ wafers, exhibits a larger blue shift (Figure 5d) than its P^+^ counterpart (Figure 5c). This greater shift is attributed to the higher oxidation susceptibility of P^++^-based structures, which leads to more extensive conversion of Si to SiO_2_, resulting in lower porosity and refractive index (see Tables 3 and 4). These trends are consistent with the higher oxide fraction and reduced Si content measured in EDS analysis (Figure 3). The porosity (P), oxidized porosity (P_ox_), oxide fraction (F_ox_), and silicon fraction (F_si_) were calculated using established equations from previous work.^1,31,32^

Recent studies have corroborated these observations. For example, Jaafar et al. demonstrated that mid-IR waveguides based on oxidized PS maintain stable refractive indices and controlled losses up to 36% oxidation, underscoring the effectiveness of dry oxidation in preserving optical performance.^63^ Meanwhile, thermally oxidized PSO_2_ structures have been shown to achieve refractive indices adjustable between **n** = 1.1 and 1.4 with high optical transparency.^64^ Finally, beam-based oxygen ion implantation, closely related to oxidation, has been successfully used to fine-tune transmission resonances in PS microcavities, demonstrating another pathway for index modulation.^65^

These hybrid structures of porous Si-SiO_2_ can be applied as either a band-stop filter or a band-pass filter. The first one could be used to block a specific range of wavelengths while allowing other wavelengths to pass. The second one could only allow a particular range of wavelengths to pass through. These filters would isolate desired spectral regions or suppress unwanted background noise in optical experiments.

Table 3 presents the refractive index, porosity (P), porosity after dry oxidation (P_ox_), oxide fraction (F_ox_), and silicon fraction (F_si_) of hybrid structures fabricated using P^+^ wafers. In contrast, Table 4 shows the corresponding values for structures fabricated using P^++^ wafers.

The values of P and P_ox_ were calculated using established relationships based on the initial and final porosities.^1,30^ As shown in both tables, the porosity after oxidation (P_ox_) is consistently lower than the initial porosity (P), which can be attributed to the growth of SiO_2_ within the pore walls. This process partially replaces air within the pores, reducing both the effective pore diameter and total porosity.

Notably, the lower silicon content in hybrid structures fabricated with P^++^ wafers results in higher oxide fractions and, consequently, greater optical transparency in the porous Si–SiO_2_ regions. This reduction in Si content diminishes optical absorption losses. However, SEM analysis of the surface layers reveals that structures fabricated with P^++^ wafers exhibit a rough surface morphology, which increases light scattering. Root-mean-square (RMS) surface roughness in porous silicon has been quantitatively linked to increased optical scattering and degraded mode quality.^66^ This surface scattering is the likely cause of the broadening and poor definition of the localized optical modes observed in Figure 5d. At shorter wavelengths, scattering losses dramatically influence the optical response of P^++^-based structures.

By contrast, the porous Si–SiO_2_ structures fabricated using P^+^ wafers show clearly defined localized modes (Figure 5c). Although these structures retain a higher silicon fraction, which could potentially contribute to absorption, the surface morphology is significantly smoother, as evidenced by SEM. Studies have shown that thermal oxidation of PS reduces RMS roughness by smoothing pore walls and reducing microscale asperities, thereby minimizing scattering losses.^53^ This smoother surface morphology is directly responsible for the improved spectral clarity and reduced optical losses observed in the P^+^-based structures.

The introduction of an asymmetric (BR)_4_-(FN)_4_-(BR)_5_ configuration is a notable contribution, as it experimentally validates theoretical predictions regarding improved field localization and transmission. To highlight this contribution, we have compared its performance to that of a symmetric equivalent, as shown in the Supporting Information. Figure S2 shows a hybrid structure of PS and porous Si–SiO_2_ obtained with a P^+^ wafer (black solid line) fabricated with a Fibonacci structure between two Bragg mirrors, which has the following symmetric sequence (BR)_5_ (FN)_4_ (BR)_5_. It is observed that when the layers number of the Bragg mirrors increased by a period, the hybrid structures were shifted to long wavelength (black solid line), but when it was subjected to two stages of dry oxidation at 350 °C for 30 min and at 800 °C for 5, 15, and 30 min (red, blue and pink dotted line), the blue wavelength shift on the transmission spectra is not shown a considerable change. It just showed an increase in the amplitude of the localized modes.

### Hybrid Structures: Absorption and Scattering Losses

The absorption loss analysis summarized in Tables 5 and 6 clearly shows the significant effect of dry oxidation in reducing optical losses in both P^+^ and P^++^ wafer-based hybrid structures. These values were obtained by normalizing the measured absorption at each localized mode’s wavelength relative to the physical thickness of the hybrid structures.

**Table 5:** Parameters to Obtain Absorption Losses in PS and Porous Si-SiO_2_ Using P^+^ Wafers

**hybrid structure**		**absorption (A.U.)**	**thickness (cm)**	**absorption losses** (cm^–**1**^ **)**
**PS**	mode 1 (747 nm)	0.0039	2.42 × 10^–04^	16.1170
	mode 2 (826 nm)	0.00071	2.42 × 10^–04^	2.9341
**porous Si-SiO**_**2**_	mode 1 (596 nm)	0.00039	2.47 × 10^–06^	1.5778
	mode 2 (659 nm)	0.00013	2.47 × 10^–06^	0.5259

**Table 6:** Parameters to Obtain Absorption Losses In PS and Porous Si-SiO_2_ Using P^++^ Wafers

**hybrid structure**		**absorption (A.U.)**	**thickness (cm)**	**absorption losses** (cm^**‑1**^ **)**
**PS**	mode 1 (747 nm)	0.00216	2.32 × 10^–4^	9.3264
	mode 2 (826 nm)	0.00053	2.32 × 10^–4^	2.2884
**porous Si-SiO**_**2**_	mode 1 (537 nm)	0.000282	2.37 × 10^–6^	1.1909
	mode 2 (595 nm)	0.000131	2.37 × 10^–6^	0.5532

In the case of P^+^ wafers (Table 5), the porous silicon (PS) structure shows relatively high absorption losses, especially at the first localized mode centered at 747 nm, with a loss of 16.12 cm^–1^, and a lower value of 2.93 cm^–1^ for the second localized mode at 826 nm. However, after the two-stage dry oxidation process, forming the porous Si-SiO_2_ hybrid, these losses are significantly reduced to 1.58 cm^–1^ at 596 nm and 0.53 cm^–1^ at 659 nm. This trend confirms that oxidation decreases the extinction coefficient, likely by reducing the density of electronic states able to absorb photons in the visible range. The substantial reduction (over 90% for the first mode) also aligns with the smoother surface morphology and the conversion of Si to SiO_2_ observed in SEM and EDS analyses.

A similar pattern appears for P^++^ wafer-based structures (Table 6), although the overall absorption loss values are generally lower than those for P^+^ before oxidation and higher after oxidation. The initial PS structure shows absorption losses of 9.33 cm^–1^ (747 nm) and 2.29 cm^–1^ (826 nm), while the oxidized version exhibits reduced losses of 1.19 cm^–1^ (596 nm) and 0.55 cm^–1^ (659 nm). These reductions are also significant, although slightly less effective than those seen in P^+^ wafers. This difference can be linked to the more aggressive oxidation of P^++^ structures, as indicated by the EDS results, which display a higher oxide content, and the SEM analysis, which shows rougher surfaces after oxidation.

The difference in postoxidation absorption losses between P^+^ and P^++^ samples is significant. In both cases, absorption at the second localized mode is lower than at the first, which may suggest that photon interaction with Si remnants is more pronounced at shorter wavelengths due to silicon’s increased absorption coefficient in the visible spectrum. Nonetheless, the better light transparency in oxidized samples highlights the effectiveness of the oxidation process in passivating the porous matrix.

These results closely match the theoretical modeling of transmission spectra and the observed shifts in localized modes. They confirm that dry oxidation not only alters the physical structure of the porous skeleton but also significantly enhances the optical quality of the hybrid structures by reducing absorption-related losses, particularly in P^+^ wafer-based systems.

Figure 6 reveals a sponge-like morphology with a silicon skeleton, where the transverse wire radius (*a*_⊥_) spans a few nanometers and the longitudinal branch size (*a*_∥_) along the growth direction reaches several tens of nanometers (see arrows in Figure 6). These structural deviations cause local fluctuations in the dielectric constant, significantly contributing to Rayleigh scattering.^32^ The values for *a*_⊥_ and *a*_∥_ were derived from SEM analyses in Figures 4 and 6.

**Figure 6:**
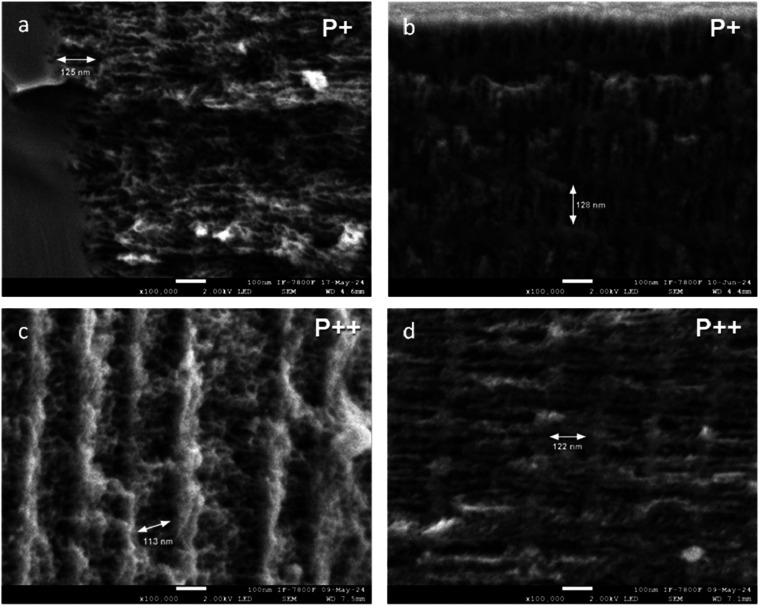
High-magnification secondary electron SEM images of the cross-section of hybrid structures fabricated with P^+^ and P^++^ wafers, before and after dry oxidation. (a, c) PS hybrid structures before oxidation, exhibiting a sponge-like Si skeleton. (b, d) Porous Si–SiO_2_ hybrid structures after two-stage dry oxidation, revealing thickened and elongated branches. Arrows indicate wire lengths along the growth direction (*a*_∥_), illustrating morphological changes due to oxidation.

Morphology varies based on the wafer type. High current-density anodization often yields branched, “cloudy” structures,^45^ an effect apparent in Figure 6c (arrow). Following dry oxidation, thin branches thicken and elongate (Figure 6b), while the porous matrix transitions into extended branches aligned with growth direction (Figure 6d). This morphological evolution impacts the optical response, as branch dimensions and wire thickness directly affect the scattered light intensity.

Using experimental *a*_⊥_ and *a*_∥_, Rayleigh scattering losses were calculated via a quantum mechanical model (Methods section of the Supporting Information)^32,52,53^ with results summarized in Tables 7 and 8. The calculations reveal that porous Si–SiO_2_ structures exhibit lower scattering at their localized modes (∼596–659 and 537–595 nm) compared to PS-only structures (747 and 826 nm).

**Table 7:** Parameters to Obtain Rayleigh Scattering Losses in PS and Porous Si-SiO_2_ Using P^+^ Wafers^a^

**hybrid structure**		***P***	**1–** ***P***	***a***_**⊥**_ **(m) Si wires with typical radius**	***a***_**∥**_ **(m) wire length**	**⟨(δε)** ^**2**^ **⟩**_***v***_ **(m** ^**3**^ **)**	**ε** ^*^	$\omega_{0}\frac{1}{s}$	$D\left(\omega_{0}\right)\left(\frac{1}{m^{3}}\right)$
**PS**	mode 1 (747 nm)	0.56	0.44	6 × 10^–9^	1.25 × 10^–7^	2.15 × 10^–21^	4.35	2.52 × 10^15^	2.17× 10^5^
	mode 2 (826 nm)	0.56	0.44	6 × 10^–9^	1.25 × 10^–7^	2.15 × 10^–21^	4.35	2.28 × 10^15^	1.77 × 10^5^
**porous Si-SiO**_**2**_	mode 1 (596 nm)	0.3	0.7	6.5 × 10^–9^	1.28 × 10^–7^	1.53 × 10^–22^	2.66	3.16 × 10^15^	1.63 × 10^5^
	mode 2 (659 nm)	0.3	0.7	6.5 × 10^–9^	1.28 × 10^–7^	1.53 × 10^–22^	2.66	2.86 × 10^15^	1.33 × 10^5^

a Where *P* is the mean porosity of the sample, *a*_⊥_ is the crystalline Si wire’s typical radius and *a*_∥_ is the typical wire length, ⟨(δε)^2^⟩_*v*_ is the volume-averaged of the porous material dielectric constant, ε* is the weighted average dielectric constant, ω_**0**_ is the angular frequency of the localized mode, and *D*(*ω*_0_) is the density of photon states.

**Table 8:** Parameters to Obtain Rayleigh Scattering Losses in PS and Porous Si-SiO_2_ Using P^++^ Wafers

**hybrid structure**		***P***	**1–** ***P***	***a***_**⊥**_ **(m) Si wires with typical radius**	***a***_**∥**_ **(m) wire length**	**⟨(δε)** ^**2**^ **⟩**_***v***_ **(m** ^**3**^ **)**	**ε** ^*^	$\omega_{0}\frac{1}{s}$	$D\left(\omega_{0}\right)\left(\frac{1}{m^{3}}\right)$
**PS**	mode 1 (747 nm)	0.54	0.46	5× 10^–9^	1.13 × 10^–7^	1.36 × 10^–21^	4.73	2.52 × 10^15^	2.46× 10^5^
	mode 2 (826 nm)	0.54	0.46	5 × 10^–9^	1.13 × 10^–7^	1.36 × 10^–21^	4.73	2.28 × 10^15^	2.01 × 10^5^
**porous Si-SiO**_**2**_	mode 1 (537 nm)	0.21	0.79	6.25 × 10^–9^	1.22 × 10^–7^	1.05 × 10^–22^	2.27	3.51 × 10^15^	1.58× 10^5^
	mode 2 (595 nm)	0.21	0.79	6.25 × 10^–9^	1.22 × 10^–7^	1.05 × 10^–22^	2.27	3.17 × 10^15^	1.29 × 10^5^

Tables 9 and 10 show that photon lifetimes are longer in oxidized structures, significantly for P^+^-based samples, confirming reduced scattering. Dry oxidation decreases losses by over 78% in P^+^ samples and by 59% in P^++^ samples. The slightly higher losses in P^++^-based samples reflect their rougher morphology (as shown by SEM) and shorter photon lifetimes at shorter wavelengths, underscoring the wavelength dependence of Rayleigh scattering.

**Table 9:** Lifetime of Photons in a PS and Porous Si-SiO_2_ Structure Using P^+^ Wafers

**hybrid structure**	**lifetime of photons in PS (ps)**	**lifetime of photons in porous Si-SiO**_**2**_ **(ps)**	**photon losses in PS** (cm^–**1**^ **)**	**photon losses in porous Si-SiO**_**2**_ **(cm** ^–**1**^ **)**
mode 1	0.012	0.054	2734	615
mode 2	0.018	0.081	1829	411

**Table 10:** Lifetime of Photons in a PS and Porous Si-SiO_2_ Structure Using P^++^ Wafers

**hybrid structure**	**lifetime of photons in PS (ps)**	**lifetime of photons in porous** Si-SiO_**2**_ **(ps)**	**photon losses in PS** (cm^–**1**^ **)**	**photon losses in porous Si-SiO**_**2**_ **(cm** ^–**1**^ **)**
mode 1	0.020	0.048	1661	689
mode 2	0.030	0.073	1111	457

## Conclusions

This work presents a comprehensive and unprecedented study of hybrid periodic and quasiperiodic structures based on PS and oxidized porous Si–SiO_2_, fabricated on both P^+^ and P^++^ doped silicon wafers with (100) orientation. While porous silicon structures and microcavities have been extensively investigated in the literature, our work is the first to systematically compare the morphological, chemical, and optical evolution of PS-based Fibonacci-like hybrid microcavities before and after dry oxidation, with a focus on substrate doping levels, porosity control, and absorption and scattering losses.

By employing a two-stage thermal oxidation process (350 and 800 °C), we successfully transformed PS into a more stable porous Si–SiO_2_ network. The oxidation not only stabilized the structure but also dramatically altered its optical behavior. Our findings show that, despite identical crystallographic orientation, the different doping concentrations in P^+^ and P^++^ wafers result in markedly different oxidation dynamics and final optical responses. This level of comparative morphological and optical analysis has not been reported in the prior literature.

Notably, the structures fabricated with P^+^ wafers showed smoother surfaces, better-preserved mesoporosity, and more sharply defined localized photonic modes after oxidation. These hybrid porous Si–SiO_2_ structures exhibited superior optical quality, with Rayleigh scattering losses reduced by over 78%, as well as improved photon lifetimes and minimal absorption due to retained silicon. Overall, the absorption loss analysis supplements the Rayleigh scattering results and reinforces the conclusion that hybrid porous Si-SiO_2_ structures have lower total optical losses than their PS counterparts, with P^+^ wafer-based designs showing the most significant improvements. This emphasizes their suitability for applications requiring low-loss photonic microcavities, such as biosensors, light-emitting devices, or optical filters. We also uniquely demonstrated, through both EDS analysis and optical modeling, the internal silicon and oxide fractions across these complex layered systems, an approach rarely reported for hybrid PS/SiO_2_ photonic systems.

Furthermore, our analysis revealed that the quasiperiodic Fibonacci structure, designed with two defect modes, provides high field localization and tunability, with mode shifts dependent on oxidation degree and porosity modulation. This result makes our structures promising candidates for tunable photonic devices, optical sensors, and low-loss microcavities.

While our study offers substantial insights, some limitations remain. The oxidation temperatures were fixed to two steps, which, although relevant for device-compatible processing, limit the exploration of graded or low-temperature oxidation regimes. Other fabrication variables (e.g., current uniformity) may influence morphology and scattering. Additionally, scattering losses were inferred through modeling rather than being measured directly with dedicated spectroscopy methods, such as photothermal deflection or ellipsometry. Further device integration and Q-factor/mode-profile analysis are necessary to evaluate the practical performance of the device.

Currently, the best Q-factor obtained with our oxidized microcavities is 113 at mode 2 (659 nm) in P^+^ wafers. It has been reported that PS microcavities fabricated in the visible spectrum region can be used as a biosensor. These results showed that the microcavities respond from 1080 to 612 nm with a quality factor of 30.^67^ Additionally, PS microcavities have been fabricated and applied as sensor devices for detecting organic solvents; the resonance peak exhibits a linear dependence on the solvent refractive index, and the quality factor decreased when the solvent refractive index increased. The quality factor values ranged from 70 to 210.^68^ However, this Q-factor would not suffice to achieve high sensitivity in the detection of hemoglobin, high-speed modulators, or lasers. More optimization of the structures would be necessary. However, they could be used for chemical sensing or as a sensor for detecting organic solvents.

Despite these constraints, our results establish a new framework for tailoring optical properties in PS/SiO_2_ hybrid structures through controlled oxidation and substrate doping. Going forward, our design could be extended to active photonic platforms by embedding optically active species such as quantum dots or oxide nanoparticles.

## Materials and Methods

### Fabrication of Hybrid Structures

First, we designed and manufactured hybrid structures from PS in the following sequence: (*BR*)^4^-(*FN*)^4^-(*BR*)^5^. The two building blocks are two layers of high and low porosity, respectively, L and H. The BRs are constructed by periodically stacking two (L and H) layers, and the recursion relation generates the FN sequences *F*_*M*_ = *F*_*M*‑1_+*F*_*M*‑2_ where *M* represents the order of the sequence (*M* = 2,3,4,···). We chose *F*_0_ = L and *F*_1_ = LH. Then, we designed an FN of the fourth order (*F*_4_ = LHLLHLHL). In this way, the porous structure can be considered a system with two defects $\left(\frac{\lambda }{2}\right)$ . Figure 7 displays the schematic illustration of an FN (*FN*)^4^ with two asymmetric Bragg mirrors ((*BR*)^4^ and (*BR*)^5^).

**Figure 7:**
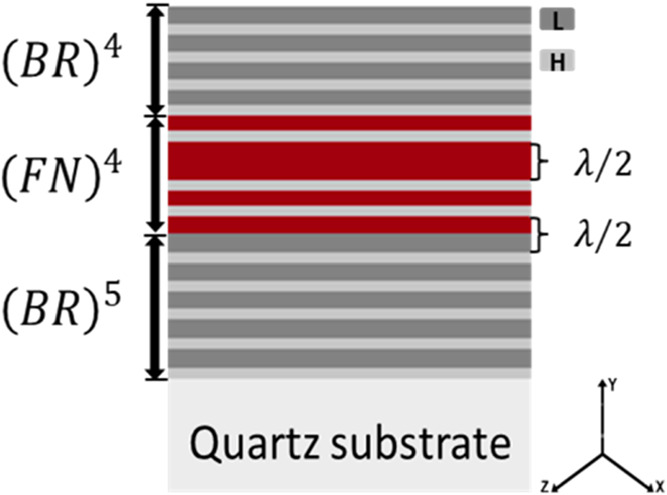
Schematic illustration of a hybrid structure.

Second, hybrid structures were obtained by electrochemical etching using a ring-shaped platinum electrode immersed in an electrolyte on silicon wafers with resistivity values of 0.01–0.02 (P^+^) or 0.001–0.005 Ω cm (P^++^), (100) orientation. The wafers were placed in a Teflon cell with an etching area of 2.011 cm^2^. An aqueous electrolyte based on HF:Ethanol with a volume ratio of 1:1 was used. A power supply (Keysight B2961A) controlled by a laptop delivers current densities to make hybrid structures. Figure 8 displays the schematic illustration of the electrochemical process and various parts of the Teflon cell.

**Figure 8:**
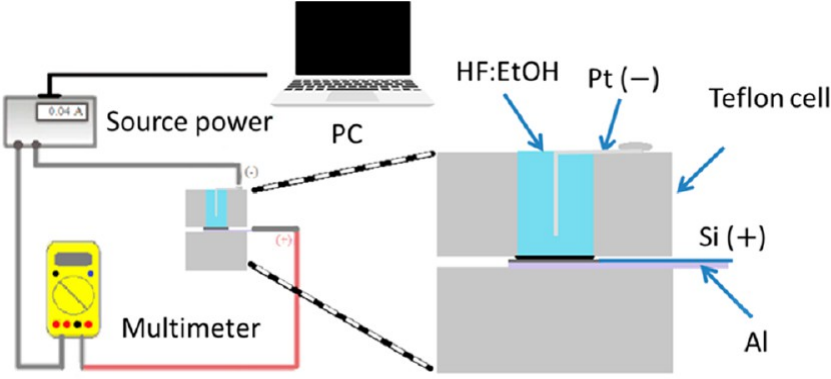
Schematic illustration of the electrochemical process and various parts of the Teflon cell.

So, two hybrid structures have been constructed by switching two current densities^69^ (80 mA/5 mA) between two values corresponding to two porosities of 66 and 46% for P^+^ wafers and 62 and 45% for P^++^ wafers; it is essential to mention that both structures were designed to respond at the same wavelength to compare them later. The current densities correspond to two refractive indices: *n*_L_ (low refractive index) and *n*_H_ (high refractive index). The anodization time to make up the first and second layers was 1.16 s (s) and 7.28 s for P^++^ wafers and 1 and 4.98 s for P^+^ wafers.

Third, hybrid structures were lifted off the Si substrate, applying a high current pulse of 550 mA for 5 s to pull off a free-standing PS membrane placed on a quartz substrate. Finally, hybrid structures on quartz substrate were oxidized by applying a two-stage dry oxidation process in an air atmosphere using an electric muffle; the first stage was based on a low-temperature treatment at 350 °C for 30 min and the second stage using a high temperature at 800 °C for 30 min, creating porous Si-SiO_2_ structures.^63,70,71^

### Optical Characterization

Transmission and absorption spectra were taken before and after dry oxidation using a Thermo Scientific Evolution 201 UV–vis spectrometer with a white-light halogen lamp serving as a source at an incidence angle of 0° degrees from 400 to 1000 nm.

### SEM and EDS Characterization

The morphology of hybrid structures, obtained with P^+^ and P^++^ wafers, was taken by a JEOL FE-SEM JSM7800 measuring the cross-section SEM (*z*-direction in Figure 6) to observe the layers and their thicknesses and the pore diameter by superficial-section backscattered electron SEM (xy-plane in Figure 6) before and after dry oxidation. The composition of both structures was obtained employing Energy-dispersive X-ray spectroscopy (EDS) before and after dry oxidation. The crystalline phase of Si wafers was measured by X-ray diffraction (XRD).

## Supplementary Material



## Notes

Optical response of PS and porous Si-SiO_2_ hybrid structures with symmetric and asymmetric Bragg mirrors and wafers’ XRD measurements (PDF)
