# Supplementary material for: Effect of Dry Oxidation on the Optical Response and Morphology of Mesoporous Hybrid Structures
Source: ACS Omega. 2025 Oct 22;10(43):50825–38. doi: 10.1021/acsomega.5c01186 (PMC12593027; doi:10.1021/acsomega.5c01186)
Supplement: Supplementary file 1 [file ao5c01186_si_001.pdf]

Supporting Information:

Optical response of PS and porous Si-SiO_2 hybrid structures with symmetric and asymmetric Bragg mirrors and wafers' XRD measurements

María R. Jiménez-Vivanco[1]*, Miller Toledo-Solano[2], Raúl Herrera[1], Maricela Santana[3], Eduardo Lugo[4,5,6,]*

[1]Instituto de Física, Universidad Nacional Autónoma de México (UNAM), Circuito de la Investigación Científica, Ciudad Universitaria, Mexico City 04510, Mexico city, Mexico; rherrera@fisica.unam.mx
[2]CONAHCYT-Facultad de Ciencias Físico-Matemáticas, Benemérita Universidad Autónoma de Puebla, Av. San Claudio y Av. 18 Sur, Col. San Manuel, Ciudad Universitaria, Puebla, 72570, Mexico
[3] Laboratorio de Biología Periodontal, División de Estudios de Posgrado e Investigación Facultad de Odontología, Universidad Nacional Autónoma de México, Mexico City, Mexico
[4] Faubert Lab, School of Optometry, University of Montreal, Montreal, QC H3T1P1, Canada.
[5] Sage-Sentinel Smart Solutions, 1919-1 Tancha, Onna-son, Kunigami-gun, Okinawa 904-0495, Japan
[6] Facultad de Ciencias Físico-Matemáticas, Ciudad Universitaria, Puebla 72570, Puebla, Mexico
* Correspondence: rayojimenezv@fisica.unam.mx (M.R.J.-V.); eduardo.lugo@sagesentinel.com

To determine the best Fibonacci structure between two Bragg mirrors of porous Si-SiO_2, we analyzed the dry oxidation effect and the role that the period number of the Bragg mirrors plays on the optical response of hybrid structures of PS and porous Si–SiO_2. Firstly, we designed various hybrid structures of PS by varying the number of periods of the Bragg mirrors and then oxidized them using a dry oxidation process. Figures S1 and S2 show that when the hybrid structures of PS are maintained and the period number of the Bragg mirrors increases (indicated by the black solid line), a red wavelength shift is observed in the transmission spectrum. This red wavelength shift can be attributed to an increase in Si in the PS structure, resulting from an increased period number in the Bragg mirrors. Additionally, Figure S1 illustrates the impact of dry oxidation on the optical response of hybrid structures comprising porous Si–SiO_2 (pink, blue, and red dotted lines), which follow the sequence $(BR)^4$ $(FN)^4$ $(BR)^5$. Here, you can observe a blue wavelength shift in the transmission spectra due to increased oxidation time from 5 to 30 minutes (min) on hybrid structures of porous Si–SiO_2. As we mention in the article, the blue wavelength shift is due to a decrease in the complex refractive index by incorporating SiO_2 in the porous matrix of PS, giving place to a reduction in the pore diameter since a Si part is replaced by SiO_2 and an air part is occupied by SiO_2.[1] In addition, you can observe a maximum blue wavelength shift of 150 nm when the PS hybrid structures were subjected to two stages of dry oxidation at 350°C for 30 minutes and at 800°C for 30 minutes (pink dotted line). As you can see, the localized modes of the porous hybrid structures of porous Si–SiO_2 are well-defined.

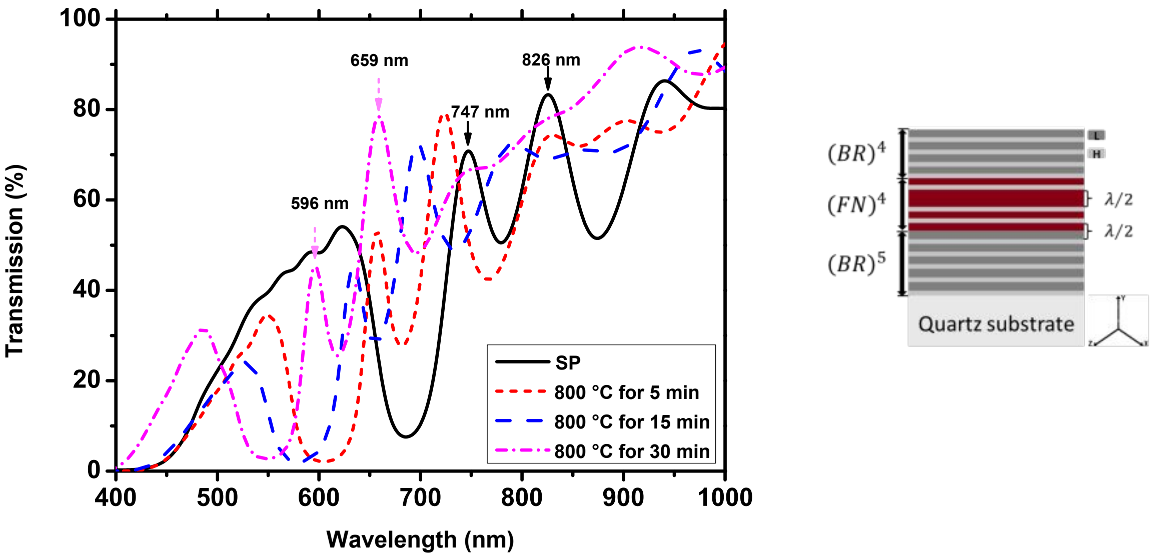

Figure S1. Experimental transmission spectra of PS (black solid line) and porous Si-SiO_2 (dotted lines) hybrid structures obtained with P+ wafers. The oxidation time applied to PS hybrid was changed from 5 to 30 min**.** All the porous structures have the following sequence $(BR)^4$ $(FN)^4$ $(BR)^5$.

Figure S2 presents a hybrid structure of PS (black solid line) fabricated with a Fibonacci sequence between two Bragg mirrors, which has the following sequence: $(BR)^{5-}$ $(FN)^{4-}$ $(BR)^5$. It is observed that when the layers number of the Bragg mirrors increased by a period, the hybrid structures of PS were shifted to long wavelength (black solid line), but when it was subjected to two stages of dry oxidation at 350°C for 30 minutes and at 800°C for 5, 15 and 30 min (red, blue and pink dotted line), the blue wavelength shift on the transmission spectra is not shown a considerable change. It just showed an increase in the amplitude of the localized modes.

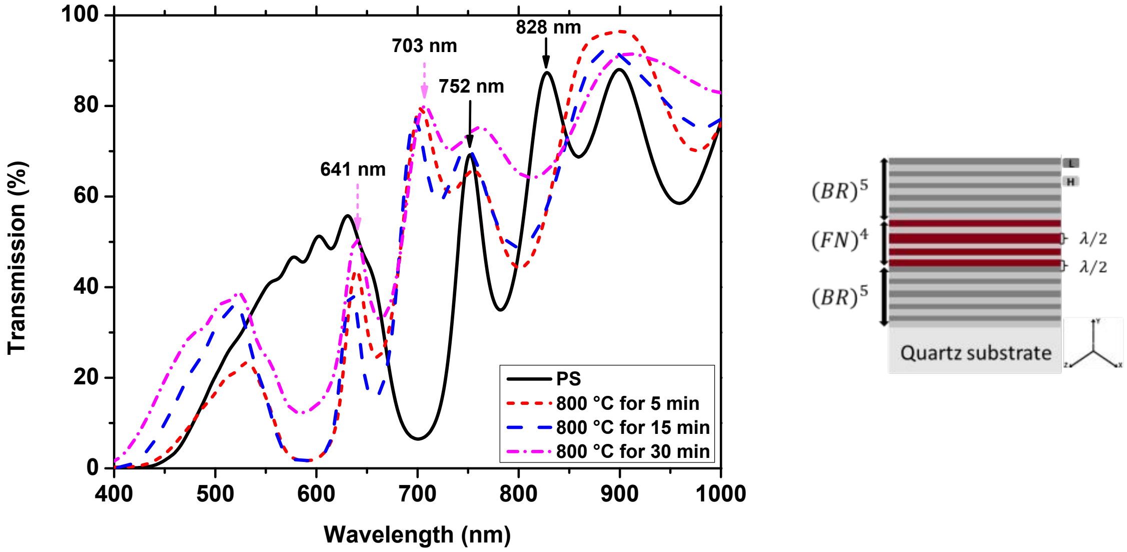

Figure S2. Experimental transmission spectra of PS (black solid line) and porous Si-SiO_2 (dotted lines) hybrid structures obtained with P+ wafers**.** The oxidation time applied to PS hybrid structures was changed from 5 to 30 min**.** All the porous structures have the following sequence: $(BR)^{5-}$ $(FN)^{4-}$ $(BR)^5$.

According to the results (Figures S1 and S2), Figure S1 showed the best optical response for the hybrid structures of porous Si–SiO_2. It was observed that localized modes were well-defined, and the maximum blue wavelength shifted to its respective position. This result is because the hybrid structures of PS with the following sequence $(BR)^4$ $(FN)^4$ $(BR)^5$ have a lower period number than the PS structures shown in Figure S2. Therefore, it is more susceptible to the dry oxidation process. The hybrid structures of PS and porous Si–SiO_2, with the sequence $(BR)^4$ $(FN)^4$ $(BR)^5$ obtained using a P+ wafer, were used to study the effect of dry oxidation on the optical response and morphology of these hybrid structures. It was compared with the Hybrid structures of porous silicon and porous Si-SiO_2 obtained with a P++ wafer.

Methods

Experimental

P^+ and P^{++} wafers XRD measurements

The X-ray diffraction analysis has been carried out on P^+ and P^{++} wafers. Figure S3 presents two peaks of intensity $2\theta = 69.15°$ that correspond to crystalline Si,[2,] confirming the (100) orientation of both Si wafers.

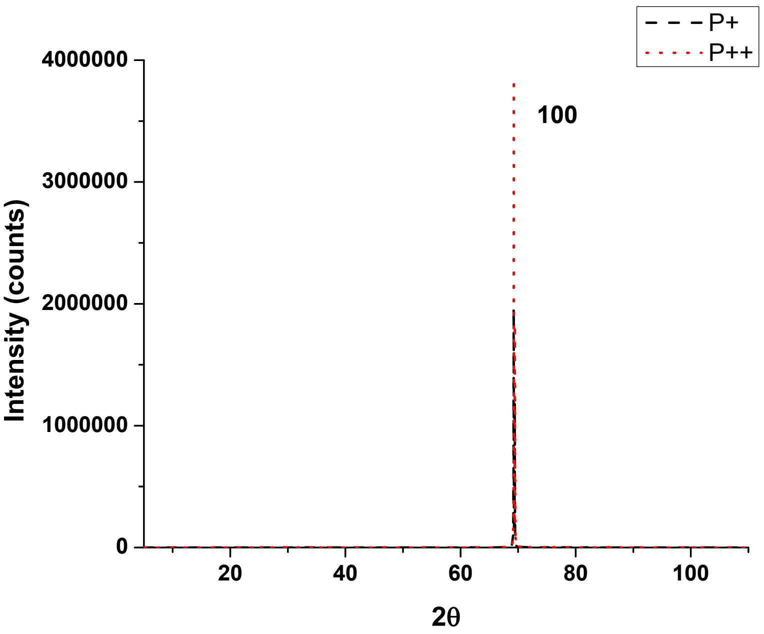

Figure S3. XRD spectra of P^+ and P^{++} wafers. In both cases, a narrow peak shows the direction of the crystallinity planes (100).

Optical losses

We made an experimental analysis of the optical losses at the wavelength of the two localized modes in hybrid structures.

The absorption losses were estimated using the absorption values at different mode wavelength positions. These values were then divided by the corresponding sample thickness in cm, resulting in the absorption coefficient.

The Rayleigh scattering analysis is achieved by a quantum mechanical model proposed by Toledo-Solano.[3,4] The model estimates the optical losses by Rayleigh scattering before and after dry oxidation at the wavelength of two localized modes in hybrid structures.

Here, the disorder in the porous structure allows for estimating the total rate of Rayleigh scattering. This model treats the features of the PS structure as a conglomeration of crystalline Si

wires with a typical radius $a_\perp$ and length $a_\parallel$ (SEM measurements obtained those); branches begin to have fluctuations and move away from a cylindrical shape.[3,4] Due to these fluctuations in the dielectric constant, the Rayleigh scattering is an important parameter as a medium of energy loss.[5] In this model, the parameters $a_{\perp\parallel}$ should be smaller than the period of the Bragg mirrors, that is, $a_{\perp\parallel} < d_H, d_L$.[4, 5]

The volume-averaged fluctuation of the PS and porous Si-SiO_2 dielectric constant is well approximated by

$$\langle(\delta\varepsilon)^2\rangle_V = 16p(1-p)(\varepsilon_2-\varepsilon_1)^2 {a_\perp}^2 a_\parallel. \tag{1}$$

where ε_1 and ε_2 are the minimal and maximum dielectric constant bounds of the porous and solid phase regions (Si and Si-SiO_2), respectively. For PS, $\varepsilon_1 = 1$ and $\varepsilon_2 = 12$, and porous Si-SiO_2 is $\varepsilon_1 = 1$ and $\varepsilon_2 = 3.9$. p is the mean porosity of the PS (P) or porous Si-SiO_2 (P_{ox}) structures.

The Rayleigh scattering losses (RSL) can be obtained as

$$\alpha_{RSL} = \frac{\Gamma_{RSL}}{c} = \frac{\pi\langle(\delta\varepsilon)^2\rangle_V {\omega_0}^2}{6{\varepsilon^*}^2 c} D(\omega_0). \tag{2}$$

where Γ_{RSL} represents the Rayleigh scattering loss rate in the hybrid structures, ω_0 is the angular frequency of the position of each localized mode, c is the speed of light within the medium, and $D(\omega_0)$ is the density of photon states in the asymmetric Bragg mirrors given by $D(\omega_0) = \frac{{\varepsilon^*}^{\frac{3}{2}} {\omega_0}^2}{\pi^2 c^3}$, which is close to the density of states in uniform media with the dielectric constant ε^* expressed as

$$\varepsilon^* = \frac{d_H\varepsilon_H + d_L\varepsilon_L}{\Lambda}. \tag{3}$$

where d_H is the high porosity layer thickness, d_L is the low porosity layer thickness, and Λ is the period ($\Lambda = d_H + d_L$), ε_H and ε_L correspond to the dielectric constants of PS or porous Si-SiO_2 layers; $\varepsilon_H = {n_H}^2$ for low porosity and $\varepsilon_L = {n_L}^2$ for high porosity layers.

In contrast, the lifetime can be defined at the wavelength of each localized mode as $\tau = {\Gamma_{RSL}}^{-1}$.

References

1. Robledo-Taboada, L. H.; Jiménez-Jarquín, J. F.; Chiñas-Castillo, F.; Méndez-Blas, A.; Camacho-López, S.; Serrano-de La Rosa, L. E.; Caballero-Caballero, M.; Alavez-Ramirez, R.; Bartolo-Alemán, M. H.; Enriquez-Porras, E. N., Tribological performance of porous silicon hydrophobic and hydrophilic surfaces. *Journal of Materials Research and Technology* **2022,** *19*, 3942-3953.
2. Cullity, B. D.; Smoluchowski, R., Elements of X-ray Diffraction. *Physics Today* **1957,** *10* (3), 50-50.
3. Toledo Solano, M.; Rubo, Y. G.; Del Río, J.; Arenas, M., Rayleigh scattering in multilayered structures of porous silicon. *physica status solidi (c)* **2005,** *2* (10), 3544-3547.
4. Rubo, Y. G.; Solano, M. T.; Del Río, J., Photon losses in porous silicon microcavities. *physica status solidi (a)* **2005,** *202* (14), 2626-2632.
5. Jiménez-Vivanco, M. R.; Herrera, R.; Martínez, L.; Morales, F.; Misaghian, K.; Toledo-Solano, M.; Lugo, J. E. In *Theoretical and Experimental Study of Optical Losses in a Periodic/Quasiperiodic Structure Based on Porous Si-SiO2*, Photonics, MDPI: 2023; p 1009.
